# Pseudarthrosis of Pelvic Fracture With Charcot Arthropathy Successfully Treated With Constrained Total Hip Arthroplasty

**DOI:** 10.7759/cureus.48295

**Published:** 2023-11-05

**Authors:** Toshiki Kitamura, Shinya Hayashi, Tomoyuki Matsumoto, Shingo Hashimoto, Naoki Nakano, Yuichi Kuroda, Takahiro Niikura, Ryosuke Kuroda

**Affiliations:** 1 Department of Orthopedic Surgery, Kobe University Graduate School of Medicine, Kobe, JPN; 2 Department of Orthopedics, Kobe University Graduate School of Medicine, Kobe, JPN

**Keywords:** constrained liner, primary syphilis, total hip arthroplasty: tha, charcot spinal arthropathy, neurogenic arthropathy

## Abstract

Charcot arthropathy is a rapidly progressive and destructive form of arthropathy caused by various neurological diseases. Total hip arthroplasty (THA) is usually contraindicated in patients with Charcot arthropathy; however, recent studies have reported good results following THA in this patient population. Herein, we report a case of Charcot arthropathy secondary to syphilis in a patient who was successfully treated with constrained THA, a new type of THA. A 56-year-old man was injured in a car accident, and a displaced acetabular fracture was revealed three weeks later. He was treated conservatively but soon developed greater displacement of the fracture and femoral head destruction. The patient was referred to our hospital for further treatment. The patient had pelvic pseudarthrosis secondary to Charcot arthropathy at the time of the first visit to our hospital. First, THA was performed with the acetabular reconstruction of the deficient bone. However, the acetabular implant was displaced one week postoperatively. THA revision was performed using a constrained cup. Postoperatively, the patient exhibited good hip stability without dislocation. However, displacement of the acetabular cup occurred one year after the second surgery. We performed a re-revision of THA using a new type of constrained cup that offers a high level of constraint to maintain range of motion (ROM) and prevent dislocations. The patient was able to walk with a T-cane one year postoperatively. Herein, we report a difficult case of revision THA in a patient with Charcot arthropathy concomitant with syphilis. THA is usually contraindicated in patients with Charcot arthropathy; however, we propose that THA using constrained cups that offer a wider ROM may be a useful therapeutic strategy for the treatment of Charcot arthropathy.

## Introduction

Charcot arthropathy, a rapidly progressive destructive arthropathy caused by various neurological diseases [1], is characterized by less pain than expected, which is attributable to the lack of sensory and nociceptive innervation in patients [2] and is diagnosed based on the radiographic appearance of bones/joints. It commonly affects the foot and ankle [3, 4], and Charcot arthropathy of the hip is rare. This condition can lead to joint destruction, bone defects, and walking difficulties. Conventionally, total hip arthroplasty (THA) is contraindicated as a therapeutic option for patients with Charcot arthropathy because many studies have reported an increased risk of complications, particularly dislocation and aseptic loosening associated with this procedure in this patient population [5]. However, recent studies have reported that the use of novel devices and innovative techniques has led to good short-term results after THA in these patients [6, 7].

Here, we report a case of Charcot arthropathy secondary to syphilis in a patient who was successfully treated with constrained THA.

## Case presentation

**Figure 1:**
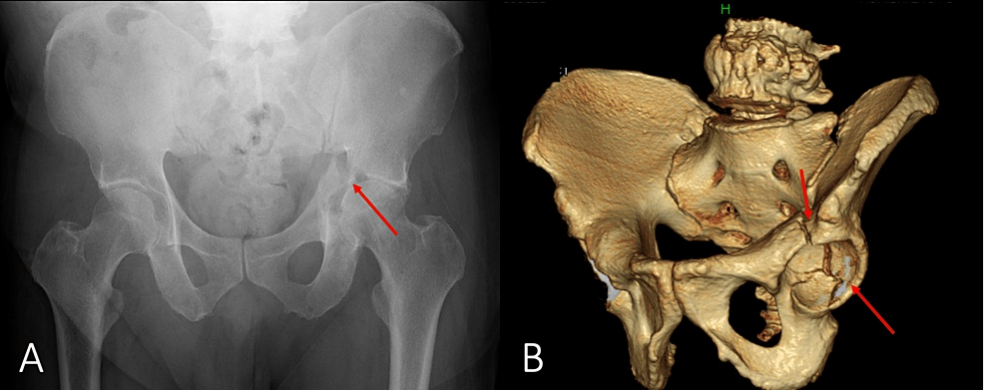
Plain radiograph and 3-D CT of the left hip obtained at the patient's primary care visit A) Frontal radiograph; B) 3-D CT of the left hip A displaced acetabular fracture is revealed (red arrows)

A 56-year-old man sustained an injury in a car accident. The patient was asymptomatic regarding the hip joint and was able to walk without difficulty. However, three weeks later, he experienced walking difficulty and visited a local primary care center. Radiography and computed tomography (CT) revealed a displaced acetabular fracture (Figure 1). 

**Figure 2:**
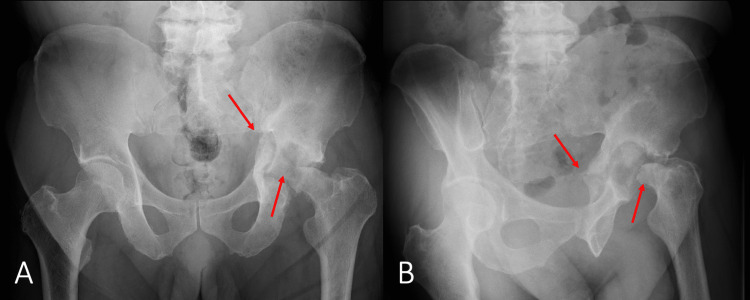
Plain radiographs of the pelvis at our hospital A) Frontal radiograph; B) Left oblique radiograph Pseudarthrosis of the transverse-posterior wall acetabular fracture and destruction of the femoral head have been demonstrated (red arrows)

**Figure 3:**
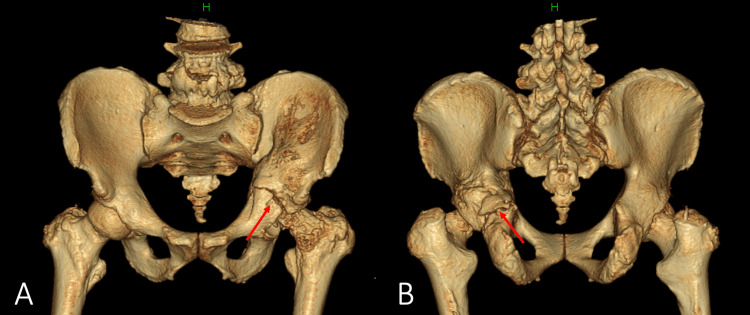
3-D CT of the pelvis at our hospital Pseudarthrosis of the transverse-posterior wall acetabular fracture and destruction of the femoral head have been demonstrated (red arrows)

A doctor at the local primary care center thought that displacement of the fracture site was minimal and selected a conservative treatment option with a non-weight-bearing status for three months. However, the patient soon developed greater displacement of the fracture and femoral head destruction and was referred to our hospital for further treatment. Radiography and CT revealed pseudarthrosis of the transverse-posterior wall acetabular fracture, with significant displacement and destruction of the femoral head (Figures 2-3).

On physical examination, we observed no hip joint tenderness; however, the patient showed a limited range of motion (ROM) for flexion and internal rotation as follows (R°/L°): extension 0°/0°, flexion 100°/50°, external rotation 45°/45°, internal rotation 40°/10°, and abduction 20°/20°. The Harris Hip Score (HHS) was 40. The sensation and muscle capacity of the lower extremities were reduced, particularly on the left side. Impaired vibration sensation in both legs was observed. The Babinski sign was bilaterally negative. Meningeal irritation was negative. Rapid plasma reagin screening and Treponema pallidum hemagglutination tests were positive. To assess for neurosyphilis, a cerebrospinal fluid (CSF) study was performed, which showed an elevated protein level of 70 mg/dL (normal: 15-60 mg/dL) and an increased white cell count of 48 cells/mm3 (normal: up to 5 cells/mm3) with lymphocytic predominance. Cerebrospinal fluid Venereal Disease Research Laboratory (CSF-VDRL) was reactive at a one-to-eight ratio. Based on the absence of constitutional symptoms, as well as the absence of pain at the fracture site, we suspected neuropathic arthropathy secondary to syphilis. 

**Figure 4:**
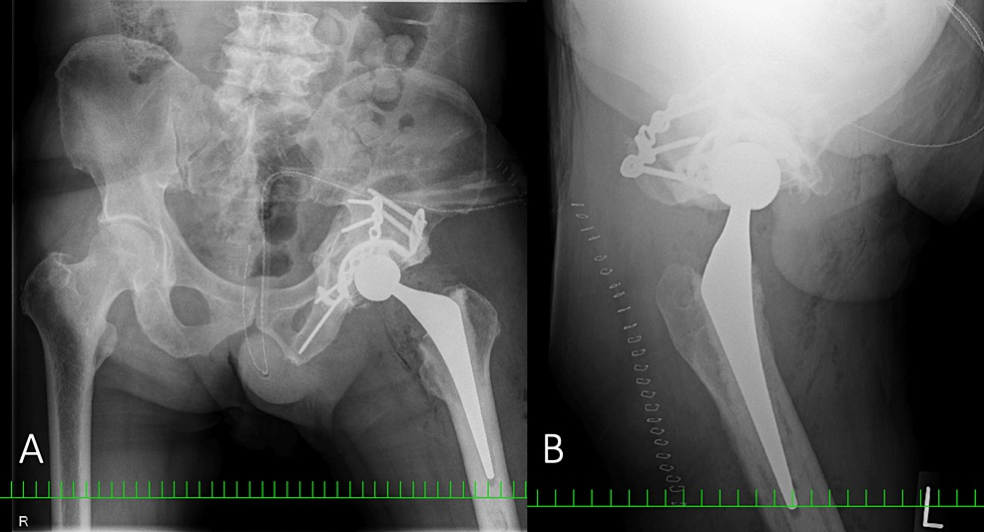
Plain radiograph after the first THA A) Frontal radiograph; B) Lateral radiograph THA - total hip arthroplasty

We performed THA with pseudarthrosis repair. The surgical procedure was as follows. We chose the direct lateral approach and detected a transverse displaced fracture in the acetabular wall. We believe that the reinforcement acetabular device may help stabilize the posterior column in addition to fixation by the reconstruction plate, and a constrained cup would limit the ROM. Therefore, the posterior column was initially fixed using a reconstruction plate (Synthes, West Chester, Pennsylvania). Acetabular reconstruction was performed using a Kerboull reinforcement acetabular device (Kyocera Medical, Kyoto, Japan) with the placement of a compacted morselized allograft, regular acetabular cup, and cemented stem (Kyocera Medical Corp., Kyoto, Japan) (Figure 4).

**Figure 5:**
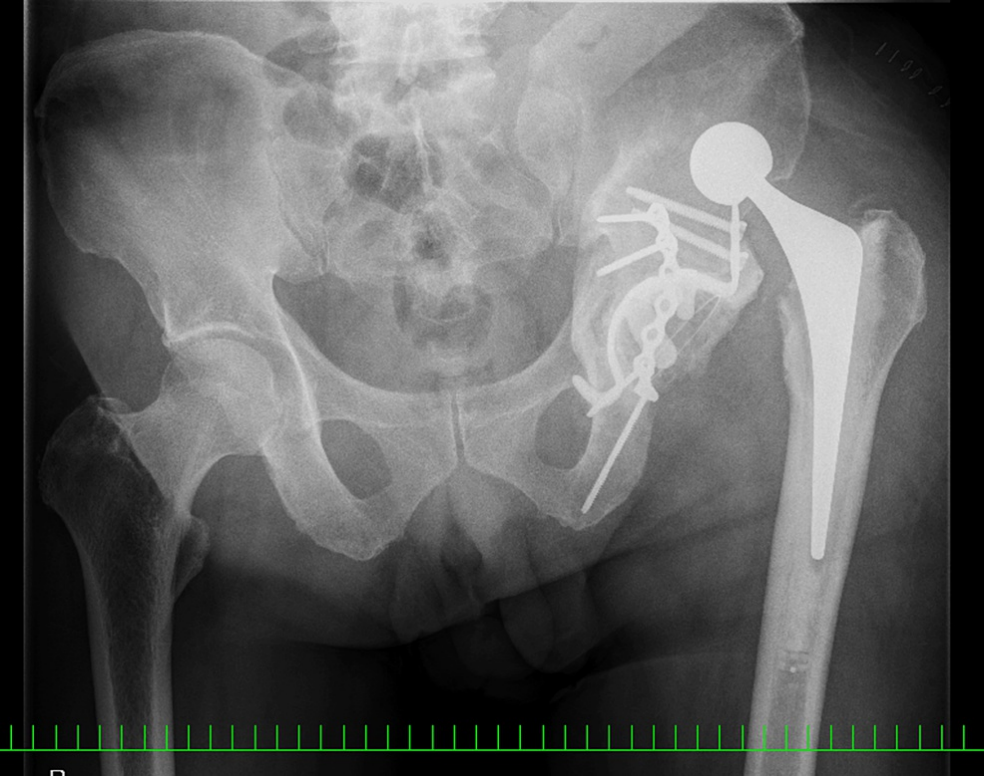
Plain radiograph one week after the first THA Posterior dislocation of the hip joint was revealed THA - total hip arthroplasty

Weight-bearing was allowed the day following surgery because the fixation of the posterior column with a combination of a reconstruction plate and reinforcement acetabular device was rigid, and the acetabular cup was stable. However, the patient developed a painless posterior dislocation of the hip joint with recurrent dislocation one week postoperatively (Figure 5).

**Figure 6:**
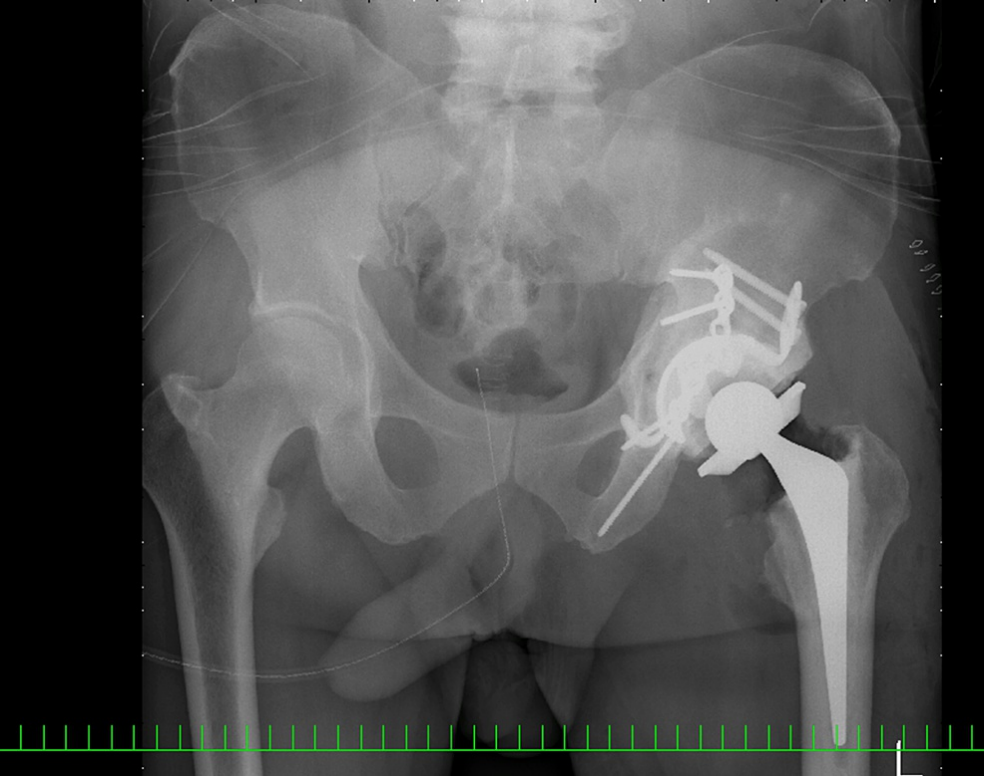
Plain radiograph after the first revision surgery

The reason for this dislocation was thought to be Weight bearing was allowed the day following surgery. Therefore, the acetabular cup was replaced with a constrained cup. We used the Trabecular Metal™ Acetabular Revision System Cemented Constrained Liner® (Zimmer Biomet, Warsaw, Indiana) on the acetabular aspect to improve hip stability (Figure 6).

**Figure 7:**
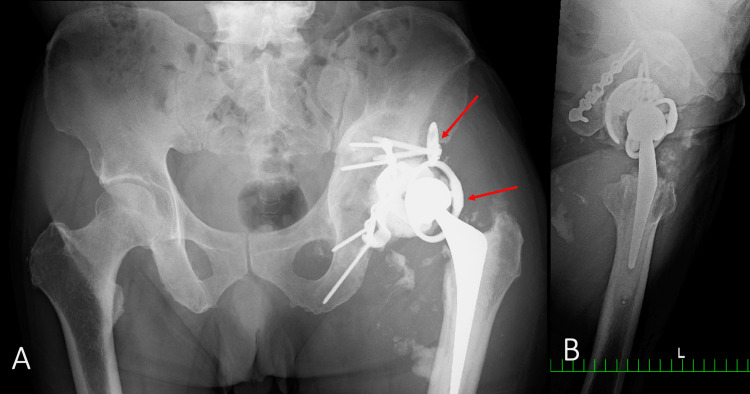
Plain radiograph one year after the second surgery A) Frontal radiograph; B) Lateral radiograph Displacement of the acetabular cup and dislocation of screws in the reconstruction plate are revealed (red arrows

Weight-bearing was allowed the day following surgery, and the patient showed good hip stability without dislocation. However, the patient experienced sudden difficulty in walking without pain one year after the second surgery. Radiography revealed displacement of the acetabular cup (Figure 7), necessitating re-revision THA. 

**Figure 8:**
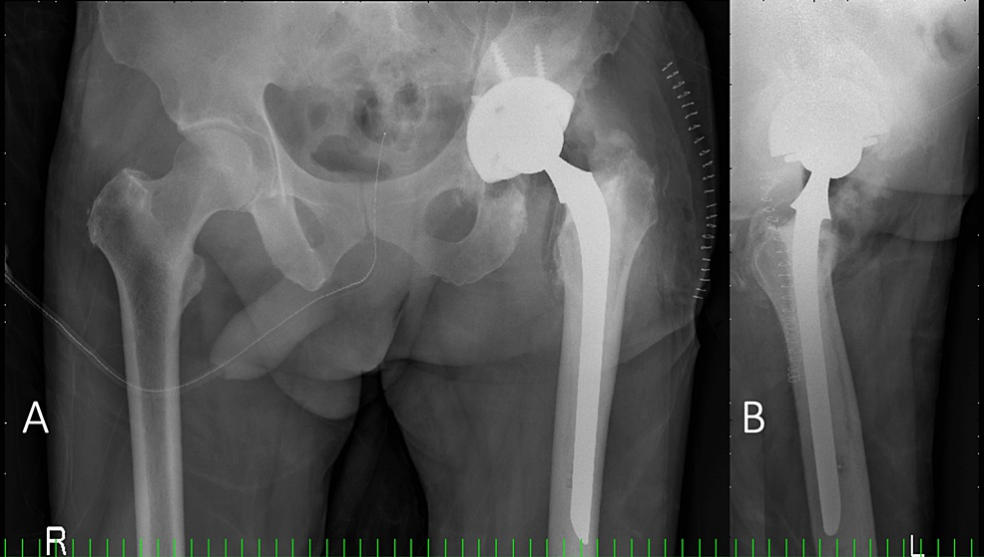
Plain radiograph after the second revision surgery A) Frontal radiograph; B) Lateral radiograph

Intraoperatively, we observed healed pseudarthrosis of the pelvic fracture; therefore, we removed all implants and placed a cementless constrained cup (G7® Freedom Constrained Liner, Zimmer Biomet, Warsaw, Indiana) and a cemented stem (CMK; Zimmer Biomet, Warsaw, Indiana) (Figure 8).

Weight bearing was introduced the day after the surgery. The patient was able to walk with a T-cane one year postoperatively with good implant survival and improved ROM as follows (R°/L°): extension 0°/0°, flexion 100°/90°, external rotation 45°/45°, internal rotation 40°/40°, and abduction 20°/20°. The HHS one year postoperatively was 86. 

## Discussion

Charcot arthropathy, also referred to as neuropathic arthropathy, was named after the neurologist Jean-Martin Charcot, who first described this condition. Charcot arthropathy is characterized by rapid progressive destruction of joints (1) and may result in bone defects, severe joint destruction, and serious malfunction. Charcot arthropathy is commonly observed in patients with diabetes mellitus, alcoholism, spinal injury, syringomyelia, syphilis, or congenital pain insensitivity.

Prior to the recent advances in and availability of effective antibiotics, Charcot arthropathy was historically associated with syphilis. Syphilitic infections cause a loss of proprioception and nociception; therefore, patients do not notice clinical symptoms during the early stages of the disease. Owing to the failure of the normal defense mechanisms of the body, patients experience recurrent microtrauma and periarticular fractures, consequently increasing bone destruction.

This neuropathic disorder predominantly affects the foot and ankle, and hip involvement is extremely rare; therefore, no treatment has been established for Charcot arthropathy of the hip [3]. Conventionally, THA is contraindicated in patients with Charcot arthropathy due to the high risk of complications, particularly dislocation, and poor clinical outcomes [5]. Brian et al. investigated complications, reoperation, revision rates, and clinical outcomes in 12 patients who underwent THA for Charcot arthropathy [8]. Patients were followed up for a mean period of five years and showed a high early complication rate (58%) and poor implant survival rate free of any revision or reoperation (67%). However, a few recent studies have reported encouraging results after THA for Charcot arthropathy of the hip [6]. Inoue et al. (2018) reported good mid-term results after THA in two patients with Charcot arthropathy of the hip. Kazimierz et al. also reported the same result in 2007 [6].

The improved post-THA outcomes in patients with Charcot arthropathy of the hip that have been reported in recent studies are attributable to technological advances in implants [9], and the availability of the constrained component is considered a useful innovation. The constrained inserts were designed to minimize the risk of dislocation [10]. Some studies have shown satisfactory results with reduced dislocation rates after THA using constrained implants [11]. However, conventional constrained inserts significantly restrict ROM, resulting in aseptic loosening and head-neck impingement [12].

A novel constrained implant that allows for greater ROM is currently available. A previous study reported favorable short-term results with a low post-THA dislocation rate (1.2%) after using this device [13]. 

A Trabecular Metal™ Acetabular Revision System Cemented Constrained Liner® (Zimmer®) was used in our patient for the first revision surgery. A constrained implant optimizes the ROM with good clinical outcomes and low dislocation and component failure rates. However, ROM did not uniformly improve across all movements; abduction and adduction were significantly restricted (48.4°) because the implant was characterized by two recesses placed at the site of impingement on the anterior-superior and posterior-inferior aspects [14]. 

We suspected that this could have caused the eversion of the acetabular cup after primary revision THA. The patient was insensitive to pain because of an underlying neurological condition. Therefore, it is reasonable to conclude that excessive movement of the hip joint (which was unnoticed owing to the absence of pain in this patient) led to the overloading of the acetabular cup with consequent eversion. 

We used the G7® Freedom Constrained Liner (Biomet®) for the second revision surgery. This component is designed to offer a high level of constraint to maintain the ROM and prevent dislocations. Karvonen et al. reported good results with this device for THA. They reported a dislocation rate of 3.8% and a mechanical failure rate of 5.7%. Compared with the Cemented Constrained Liner® (Zimmer®), the G7® Freedom device offers a wider ROM (55° of adduction and abduction, 35° of internal and external rotation, and 140° of flexion and extension) [15]. Successful THA performed for Charcot arthropathy of the hip in our patient was attributed to the wide range of ROM offered by the device.

This study has some limitations in terms of short-term evaluation. Long-term clinical and radiographic follow-ups are required.

## Conclusions

Herein, we report a difficult case of revision THA in a patient with Charcot arthropathy concomitant with syphilis. We propose that THA using constrained cups, which offer a wider ROM, may be a useful therapeutic strategy for Charcot arthropathy.
